# Biomimetic Spiral-Reinforced Honeycomb for Integrated Energy Absorption Under Complex Loading Scenarios

**DOI:** 10.3390/biomimetics11040277

**Published:** 2026-04-17

**Authors:** Junhao Nian, Zhenyu Huang, Yingsong Zhao, Kai Liu

**Affiliations:** 1 School of Traffic & Transportation Engineering, Central South University, Changsha 410083, China; 2 National Key Laboratory of Solid Rocket Propulsion, Institute of Xi’an Aerospace Solid Propulsion Technology, Xi’an 710025, China; 3 Frontiers Science Center for Extreme Flows and Energies, Central South University, Changsha 410083, China

**Keywords:** energy-absorbing materials, biomimetic design, spiral structure, SEA

## Abstract

Planar honeycomb structures, especially biomimetic hexagonal honeycombs, are widely used in energy-absorbing equipment because of their excellent out-of-plane deformation resistance. However, their significant mechanical anisotropy, manifested by the large discrepancy between out-of-plane and in-plane responses, greatly restricts their broader applications. Inspired by spiral-reinforced thin-walled biological tubular systems, such as animal tracheae and plant vessels, this study proposes a biomimetic reinforcement strategy by embedding spiral structures along the thin walls of planar honeycombs. To validate the feasibility of the proposed design, biomimetic honeycomb specimens were fabricated using 3D-printing technology and tested under compression along different loading directions. Furthermore, a numerical model validated against the experiments was developed to reveal the underlying enhancement mechanism. The results demonstrate that the proposed biomimetic honeycomb preserves the favorable out-of-plane performance of the conventional hexagonal honeycomb, while improving the in-plane energy absorption capacity by up to 70%. The biomimetic spiral reinforcements enable more effective load transfer under multidirectional loading, resulting in a more uniform plastic stress distribution over the entire structure and activating a larger deformation region for energy dissipation. The present work provides a bioinspired strategy for developing lightweight energy-absorbing structures for potential applications in aerospace, rail vehicles, marine engineering, and civil structures.

## 1. Introduction

Honeycomb structures have been widely recognized as lightweight cellular architectures with high specific stiffness, high specific strength, and excellent out-of-plane deformation resistance and energy absorption capability [1]. Owing to these merits, they have been extensively used in sandwich cores [2], buffering components [3], and lightweight load-bearing modules for crashworthiness [4], blast resistance [5], and impact protection in aerospace [6], transportation [7], marine [8] and civil fields [9]. In practical service, such structures are often required not only to sustain normal compressive loads, but also to dissipate impact energy and maintain structural integrity under complex loading conditions. However, conventional planar honeycombs exhibit pronounced mechanical anisotropy [10]. Their strong out-of-plane performance is typically dominated by progressive buckling, whereas the in-plane response is much weaker because the thin walls are dominated by bending-dominated deformation and localized instability [11]. This large discrepancy between out-of-plane and in-plane mechanical behavior severely constrains the applicability of conventional honeycombs in multidirectional loading environments, e.g., the loading path is uncertain or changes during service. Therefore, enhancing the integrated energy absorption capacity of honeycomb structures under complex loading scenarios has become a key issue in the design of high-performance lightweight protective structures.

To address this issue, numerous structural optimization strategies have been developed. Typical approaches include modifying the cell topology, introducing hierarchical or graded architectures [12], embedding auxiliary substructures [13], and adding reinforcing struts or hybrid features [14]. These strategies can improve the in-plane stiffness, alter the deformation mode, and increase the specific energy absorption of conventional honeycombs to different extents. Recently, origami-embedded honeycombs have shown that embedding additional structural motifs into conventional honeycomb walls can effectively weaken anisotropy and promote more comparable energy absorption in multiple directions [15]. Nevertheless, most existing designs still focus on geometric modification or local strengthening, and only a limited number of studies have aimed at achieving a more balanced enhancement of energy absorption in both out-of-plane and in-plane directions through a clear reinforcing mechanism.

In recent years, biomimetic design has emerged as an effective route for improving the mechanical performance of cellular materials and sandwich structures [16]. Natural biological systems often exhibit multifunctionality, structural hierarchy, and efficient load-transfer pathways, which provide valuable guidance for engineering design [17]. Existing bioinspired energy-absorbing structures have drawn inspiration from bamboo [18,19], pomelo peel [20], spider webs [21], mantis shrimp appendages [22], and other biological architectures [23,24], demonstrating clear potential for improving impact resistance, toughness, and crushing efficiency. And, a beetle elytron-inspired structure, which is representative in terms of curved configuration and compression mechanism analysis [25]. In addition, helical or spiral reinforcement methods widely observed in biological tubular systems are of special interest. For example, plant tracheary elements and xylem vessels develop helical wall thickenings that help the conduit withstand negative pressure while maintaining flexibility [26], and biomechanical analyses have shown that these morphologies follow efficient engineering design principles [27]. Biological porous structures provide a rich source of inspiration for creating lightweight architectures with improved energy absorption, especially when combined with additive manufacturing.

Nevertheless, a biomimetic strategy that simultaneously preserves out-of-plane performance and improves in-plane energy absorption through a clear reinforcing mechanism remains underexplored. Motivated by these considerations, this study proposes a biomimetic spiral-reinforced honeycomb by integrating spiral substructures into the thin walls of a conventional planar honeycomb. The basic idea is to mimic the reinforcing role of helical patterns in natural systems and use the embedded spiral pathways to enhance load transfer and deformation coordination under different compression directions [28]. In this way, the proposed structure is expected to retain the favorable out-of-plane crushing characteristics of the traditional honeycomb while substantially improving its weak in-plane energy absorption performance. To validate the feasibility of this design, biomimetic spiral-reinforced honeycomb specimens were fabricated by 3D printing and tested under quasi-static compression along different directions. An experimentally validated finite element model was then established to investigate the effects of geometric parameters and to reveal the underlying deformation and energy-dissipation mechanisms. This work is organized as follows: Section 2 presents the biomimetic concept and geometric design of the spiral-reinforced honeycomb. Section 3 describes the fabrication process and quasi-static compression experiments. Section 4 develops the finite element model. Section 5 discusses the enhancement effect and the associated mechanisms of the proposed structure. Finally, the main conclusions are summarized in Section 6. This work provides a new bioinspired strategy for balancing the multidirectional energy absorption of honeycomb structures and may contribute to the development of lightweight protective components for aerospace, rail vehicles, marine engineering, and civil infrastructure.

## 2. Design of the Biomimetic Spiral-Reinforced Honeycomb Structure

### 2.1. Biomimetic Design Principle

During long-term biological evolution, tubular functional structures in organisms have often needed to withstand complex multidirectional and varying loads. Spiral-reinforced configurations are therefore widely found in functional tissues across different species, showing excellent adaptability to such complex mechanical environments. While maintaining the deformation capability required for biological functions, these structures can effectively resist collapse induced by multidirectional loading. This provides a natural prototype for the biomimetic design of engineering energy-absorbing structures.

**Figure 1:**
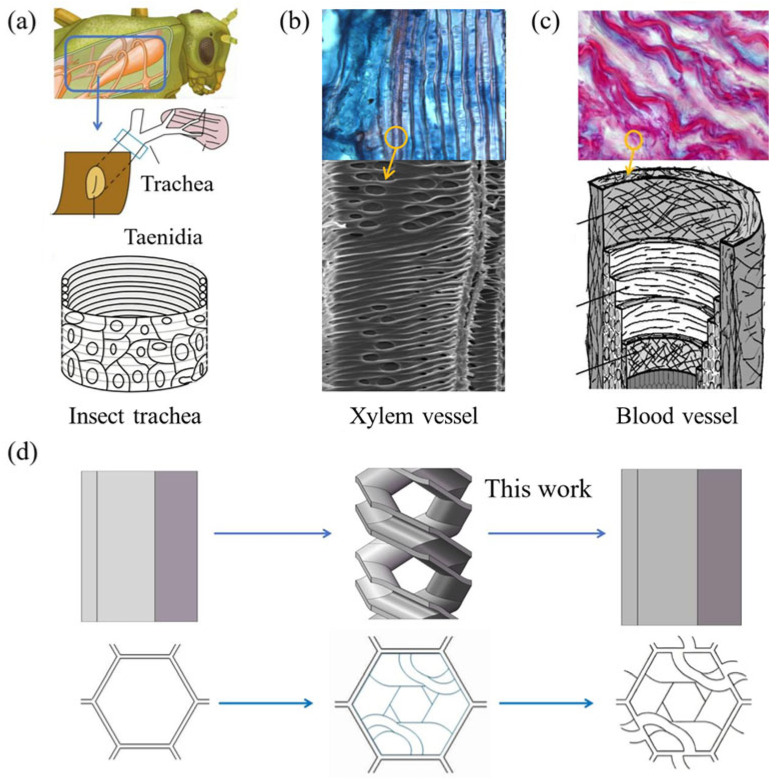
Biological prototypes and design concept of the biomimetic spiral-reinforced honeycomb structure: (**a**) spiral supporting structure in insect tracheae [29]; (**b**) helical thickening structure in plant vessels [30]; (**c**) bidirectional helical collagen architecture in the vascular tunica media; and (**d**) design process of the proposed biomimetic structure [31]. Arrows indicate the key structural features; blue boxes highlight the corresponding biological prototypes.

As shown in Figure 1a, the insect trachea is a key component of the respiratory system [29]. During crawling, flying, and other body movements, the trachea undergoes stretching and bending deformation together with the body, while also being subjected to multidirectional compression from the surrounding muscles and body-wall tissues. The spiral thickening structures distributed along the inner wall, namely the taenidia, can effectively reduce the risks of radial collapse under multidirectional loading. As shown in Figure 1b, the vessels in the primary xylem are the core structures responsible for water transport in plants [30]. During plant growth, wind-induced swaying, and external squeezing by the surrounding environment, these vessels are subjected to coupled axial tension and radial compression, resulting in a highly complex and variable loading. The helical secondary wall thickenings in the cell wall can effectively disperse the applied loads through their spiral configuration, thereby preventing conduit collapse under complex loading conditions. As shown in Figure 1c, collagen fibers in the tunica media of mammalian blood vessels are arranged in a bidirectional helical manner [31]. During physiological activity, blood vessels are not only subjected to periodic pulsating internal pressure caused by heartbeat, but also experience bending, torsion, and stretching associated with body movement, thus facing multidimensional loading. This bidirectional helical fiber arrangement enables the vessel wall to simultaneously sustain circumferential, axial, and torsional loads, thereby significantly enhancing its structural stability under complex loading.

In summary, these natural structures reveal a universal mechanical principle: spiral-reinforced configurations endow tubular systems with exceptional adaptability to complex and multidirectional loads. Specifically, the spiral topology achieves three core functional benefits simultaneously: (a) it effectively disperses concentrated loads across the structure, avoiding localized stress concentration and catastrophic collapse; (b) it maintains sufficient structural flexibility to meet the deformation requirements of biological functions; and (c) it introduces additional energy-dissipation paths through progressive plastic deformation of the spiral components, thereby significantly improving the overall energy absorption capacity. This fundamental rationale provides a clear biological inspiration for the design of high-performance engineering energy-absorbing structures. Based on this biomimetic principle, this study takes the conventional hexagonal honeycomb cell as the baseline structure and integrates the spiral-reinforced configuration observed in the above biological tissues into the cell walls of the hexagonal honeycomb, as shown in Figure 1d. In addition, compared with a single-spiral configuration, the double-spiral arrangement provides point-symmetric load-transfer pathways within each cell, enabling more balanced reinforcement under both in-plane loading directions and avoiding the directional bias that a single spiral would introduce.

### 2.2. Configuration of the Biomimetic Spiral-Reinforced Honeycomb

**Figure 2:**
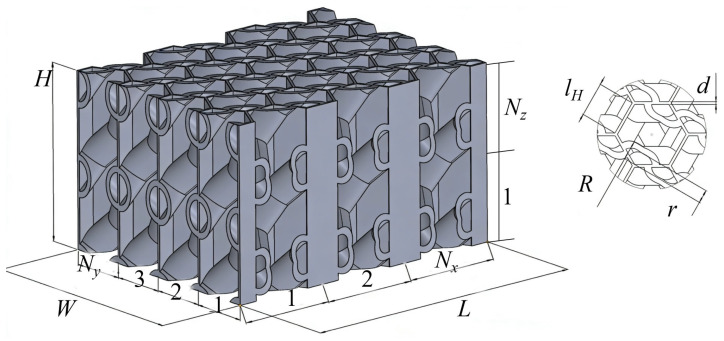
Topological configuration of the biomimetic spiral-reinforced honeycomb.

According to the above design principle, a honeycomb structure with embedded spiral reinforcements was designed, as shown in Figure 2. The overall length, width, and height of the honeycomb are denoted by L, W, and H, respectively. The structure contains $N_{x}$ periodic unit cells along the length direction and $N_{y}$ periodic unit cells along the width direction, and $N_{z}$ periodic unit cells along the height direction. The wall length and wall thickness of the proposed honeycomb are denoted by $l_{H}$ and d, respectively.

The spiral reinforcement has a semicircular cross-section, with an inner radius of r and an outer radius of R. Accordingly, the wall thickness of the spiral structure is $d_{L}=R-r$. In this study, to specifically investigate the reinforcing effect induced by structural features, the wall thickness of the spiral reinforcement was designed to be identical to that of the honeycomb walls. In addition, its mean radius was set to half of the honeycomb wall length, i.e., $l_{H}/2=(R+r)/2$.

To achieve a stronger spiral-reinforcement effect within a limited structural height, two spiral reinforcements were introduced into each honeycomb cell, as shown in Figure 1d. In the plane of the honeycomb, namely the in-plane direction, these two spirals are arranged in a centrosymmetric manner. Moreover, to avoid interference between the two spiral reinforcements, the helical inclination was selected as an intermediate value between 0° and 90°, namely a helix angle of 45°. Therefore, the main design parameters of the proposed structure include the wall length and wall thickness of the honeycomb, as well as the overall length, width, and height of the structure.

It should be particularly noted that, for comparative analysis, a conventional hexagonal honeycomb was correspondingly established for each spiral-reinforced configuration with a given set of structural parameters. The conventional honeycomb had the same overall length, width, height, and wall length as the proposed biomimetic structure. However, to ensure the same total mass, the wall thickness of the conventional hexagonal honeycomb, denoted by $d_{H}$, was correspondingly increased, since the introduction of the spiral reinforcements increased the total mass of the proposed structure.

## 3. Experimental Procedures

### 3.1. Experimental Specimens and Test Setup

The experimental specimens were manufactured by fused deposition modeling (FDM) 3D printing using polylactic acid (PLA). For the proposed biomimetic spiral-reinforced honeycomb, the inner and outer radii of the spiral reinforcement were set as r=9 mm and R=11 mm, respectively. The wall length and wall thickness of the honeycomb were $l_{H}=20\;\mathrm{mm}$ and d=2 mm, respectively. The numbers of periodic unit cells along the length and width directions were $N_{x}=3$ and $N_{y}=4$, respectively. Accordingly, the overall dimensions of the specimen were L=180 mm, W=138.5 mm, and H=120 mm. To ensure the repeatability of the experimental results, two identical specimens were fabricated and tested under the same conditions.

**Figure 3:**
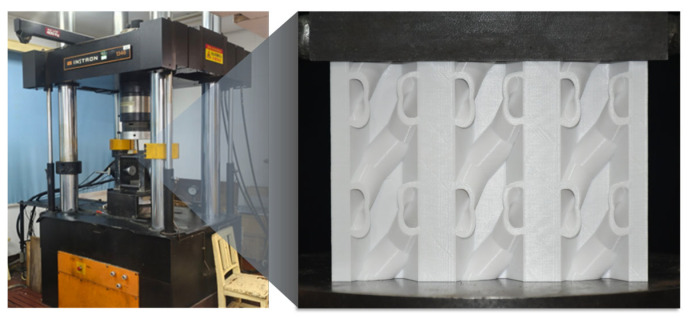
Overview of the fabricated specimens and the INSTRON 1346 testing system.

As shown in Figure 3, the quasi-static compression tests were carried out using an INSTRON 1346 mechanical testing system. The specimens were placed on the loading platform, and the upper platen moved downward along the Z-direction at a constant speed of 4 mm/min to apply the compressive load. The force–displacement data were collected automatically by the sensors of the testing system, with an acquisition frequency of 5 Hz. The maximum quasi-static load capacity of the INSTRON 1346 system is 2000 kN, and the load measurement accuracy is ±0.5%. During the test, the deformation process of the specimen was recorded simultaneously for subsequent observation of the crushing mode and failure characteristics.

### 3.2. Experimental Test Process and Results

**Figure 4:**
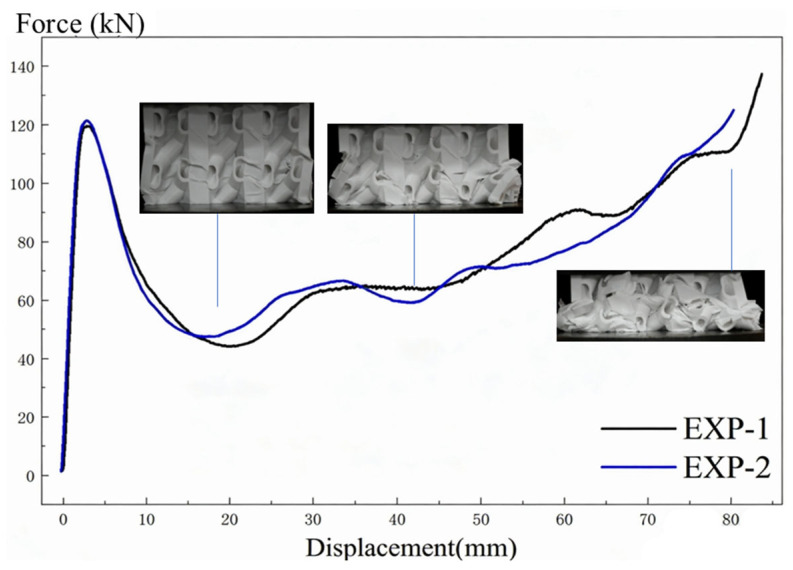
Force–displacement curves and deformation sequences of the biomimetic honeycomb under quasi-static compression.

Figure 4 shows the force–displacement curves and typical deformation modes of the biomimetic spiral-reinforced structure under quasi-static compression. The two repeated tests show good consistency in both peak force and subsequent crushing response. The first peak force appears at a displacement of about 4–5 mm, with a value close to 120 kN. After that, the force drops rapidly to about 45–50 kN, indicating the initiation of local instability and collapse. With further compression, the structure enters a relatively stable progressive collapse stage, in which the force fluctuates within the range of about 60–90 kN over a large displacement interval. During this stage, the cell walls and spiral substructures deform coordinately, and the collapse gradually propagates from the local region to the whole specimen. Finally, when the displacement exceeds about 70 mm, the structure enters the densification stage, and the force rises rapidly again. These results indicate that the proposed structure can maintain a relatively stable load-bearing capacity during progressive crushing and long plateau (above 65%), which is beneficial for enhanced energy absorption. In this work, although only two specimens were tested per condition due to material and fabrication constraints, the two repeated tests show good consistency in both peak force and plateau response, with a coefficient of variation of less than 0.76% in peak force.

## 4. Simulation Method

### 4.1. Material Constitutive Model and Parameter Determination

**Figure 5:**
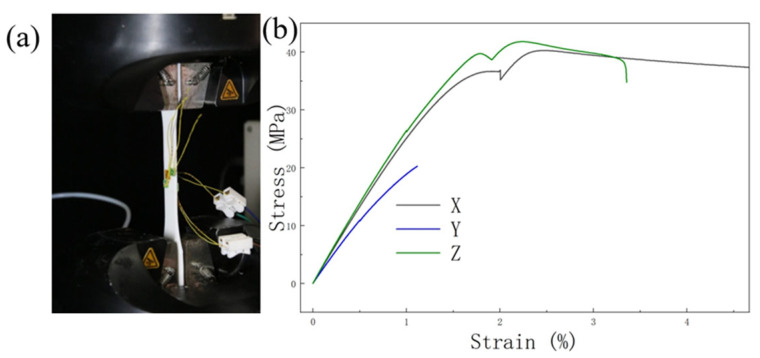
Testing of anisotropic material properties of PLA. (**a**) Tensile test of mechanical properties of 3D-printed PLA specimens; (**b**) tensile stress–strain curves along X, Y, and Z printing directions.

In the numerical simulation, an elastic-perfectly plastic constitutive model was adopted for the PLA material. To determine the corresponding material parameters, tensile tests were carried out on 3D-printed PLA specimens with different printing orientations by following the standard mechanical testing procedure, as shown in Figure 5. According to the tensile stress–strain curves, the elastic modulus, yield strength, and other basic mechanical parameters in different printing directions were obtained.

It should be noted that the proposed biomimetic spiral-reinforced honeycomb contains structural features distributed in multiple directions, and the local printing paths in the fabricated specimen are not limited to a single orientation. Therefore, to reasonably represent the overall material response of the printed structure, the average values of the measured material parameters in different printing directions were used in the present simulation. The detailed material parameters adopted in the constitutive model are listed in Table 1.

### 4.2. Simulation Model and Validation

**Figure 6:**
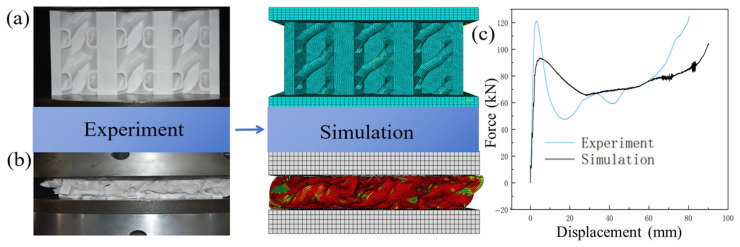
Comparison of experimental and simulation results. (**a**) Initial compression state; (**b**) compressed dense state; (**c**) comparison of force–displacement curves between experiment and simulation.

A finite element model was established in ABAQUS/Standard to simulate the quasi-static compression behavior of the PLA biomimetic spiral-reinforced honeycomb and the conventional hexagonal honeycomb, with boundary conditions fully consistent with the quasi-static compression tests. The numerical setup was designed to match the experimental loading mode, support constraints, ensuring reliable comparison and validation between simulation and test results. As shown in Figure 6, the experimental loading and supporting platens were modeled as two square rigid plates with a side length of 200 mm and a height of 20 mm. The lower plate was fully fixed, while the upper plate was imposed with a prescribed translational motion along the Z-direction at a constant speed of 4 mm/min. The reaction force and displacement at the reference point of the loading plate were extracted to construct the force–displacement response.

The thin-walled structure was modeled by shell elements and discretized using a quadrilateral-dominated mesh to ensure stable and continuous meshing in the complex spiral regions and to maintain sufficient mesh quality for the subsequent analysis. Shell elements are acceptable for the present geometry because the wall thickness-to-characteristic-length ratio of both the honeycomb walls and spiral reinforcements is small, satisfying the thin-shell assumption. Although the curved spiral regions introduce geometric complexity, the quadrilateral-dominated mesh with refined discretization ensures adequate representation of the local curvature, and the convergence analysis confirmed that the 2 mm element size yields mesh-independent results. Then, the contact between the thin-walled structure was defined as hard contact, and the friction coefficient was taken as 0.14.

The validity of the model was tested by comparing the numerical results with the experimental results. As shown in Figure 6c, the simulated force–displacement curve agrees well with the experimental one in the overall variation trend. In particular, the simulation gives reasonable predictions for the initial stiffness, the collapse plateau, and the onset displacement of densification. Due to the geometric imperfections introduced during specimen fabrication, the material inhomogeneity of the printed PLA, and the simplified equivalent shell modeling in the simulation, some deviations still exist in local intervals. The simulation slightly overestimates the plateau force, with a mean absolute percentage error of approximately 12% over the crushing plateau region. This deviation is considered acceptable for quasi-static honeycomb simulations and is consistent with the simplifications inherent in the elastic-perfectly plastic constitutive model and the equivalent shell representation of the printed geometry.

Under the same compression displacement, the simulated collapse pattern is generally consistent with the experimental observation. It indicates that the present model can reasonably capture the actual compression behavior of the biomimetic spiral-reinforced honeycomb. Therefore, it was used for the subsequent simulation analysis and for further investigation of the energy-absorption mechanism. In addition, it should be noted that, to simplify the numerical simulation, an elastic-perfectly plastic constitutive model was adopted in this study, and the slight post-yield hardening observed in the PLA tensile curves was neglected. This simplification was considered acceptable in this work as shown in Figure 6c. However, a more refined constitutive model including post-yield hardening should be considered in future work for higher-accuracy prediction.

## 5. Results and Discussion

This section discusses the mechanical response of the proposed biomimetic spiral-reinforced honeycomb under different loading directions and discusses the corresponding enhancement mechanism. Firstly, the out-of-plane compression behavior is examined to clarify whether the introduced spiral reinforcements preserve the crushing characteristics of the conventional honeycomb. Then, the in-plane compression performance is analyzed to evaluate the improvement in the weak loading direction after spiral reinforcement. Finally, the deformation patterns, load-transfer characteristics, and plastic region distribution are further discussed to reveal the underlying mechanism by which the biomimetic spiral design enhances the integrated energy absorption performance of the conventional honeycomb structure.

### 5.1. Favorable Out-of-Plane Performance

To clarify whether the proposed biomimetic spiral-reinforced design can improve the integrated mechanical performance of the honeycomb without sacrificing its original out-of-plane advantage, the out-of-plane energy-absorption behavior was first investigated. This is because many existing strategies for improving the multidirectional performance of conventional honeycombs often weaken the original out-of-plane crushing resistance. For honeycomb structures, the wall thickness-to-length ratio is one of the key geometric parameters governing the mechanical response. In this study, the wall thickness of the biomimetic honeycomb was set to 2 mm, which is a relatively mature and reliable thickness for FDM 3D printing, while the wall length was increased from the experimental baseline of 20 mm to 22.5 mm, 25 mm, 27.5 mm, and 30 mm. For each biomimetic configuration, a conventional hexagonal honeycomb with the same mass, volume, and wall length was established for comparison. Accordingly, the wall thickness of the conventional honeycomb was slightly increased, since the proposed biomimetic structure contains added spiral-reinforcement parts.

**Figure 7:**
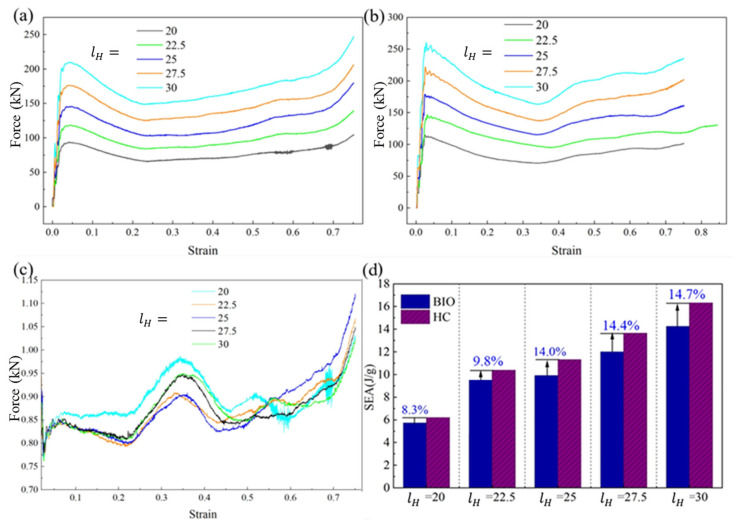
Favorable out-of-plane crushing behavior. (**a**) Response curves of the biomimetic honeycomb with different wall lengths (or different thickness-to-length ratios). (**b**) Response curves of the conventional honeycomb with the same mass and volume as the biomimetic honeycomb. (**c**) Ratio of the compressive force of the biomimetic honeycomb to that of the conventional honeycomb. (**d**) Comparison of the out-of-plane specific energy absorption (SEA) between the biomimetic honeycomb and the conventional one.

Figure 7a,b compares the out-of-plane compressive responses of the biomimetic honeycombs and the corresponding conventional honeycombs, respectively. It can be seen that both structures exhibit a similar overall response pattern, including a sharp initial peak, a subsequent load drop, a relatively stable crushing stage, and a final force rise associated with densification. A direct comparison between Figure 7a,b further shows that the conventional honeycomb generally exhibits a slightly higher force level than the corresponding biomimetic honeycomb under the same mass and volume. Under out-of-plane compression, the conventional honeycomb benefits more directly from the increased wall thickness, whereas part of the material in the biomimetic structure is distributed into the embedded spiral parts. Even so, the difference between the two structures remains limited over most of the crushing process. As shown in Figure 7c, the ratio of the compressive force of the biomimetic honeycomb to that of the conventional honeycomb remains close to 1.0 and is mostly maintained within a narrow range of about 0.85–0.95. This indicates that the introduced spiral reinforcement does not cause a substantial loss of out-of-plane crushing resistance.

The same conclusion can be drawn from the specific energy absorption (SEA) shown in Figure 7d, where SEA was calculated as the external work input before densification, which was obtained from the compressive force response curve, and the densification point was identified as the last valley point on the response curve. For both structures, the out-of-plane SEA increases gradually with increasing wall length. More importantly, the SEA of the biomimetic honeycomb remains highly comparable to that of the conventional honeycomb over the whole parameter range. The relative difference is only 8.3% at $l_{H}=20$ mm. In other words, although the proposed structure introduces additional spiral-reinforcement parts, its out-of-plane energy absorption is still maintained at a level close to that of the conventional honeycomb. This is important for the practical application of the proposed structure because it means that the multidirectional performance of the conventional honeycomb can be enhanced without paying a substantial penalty in its original dominant loading direction.

### 5.2. Significant In-Plane Performance Improvement

**Figure 8:**
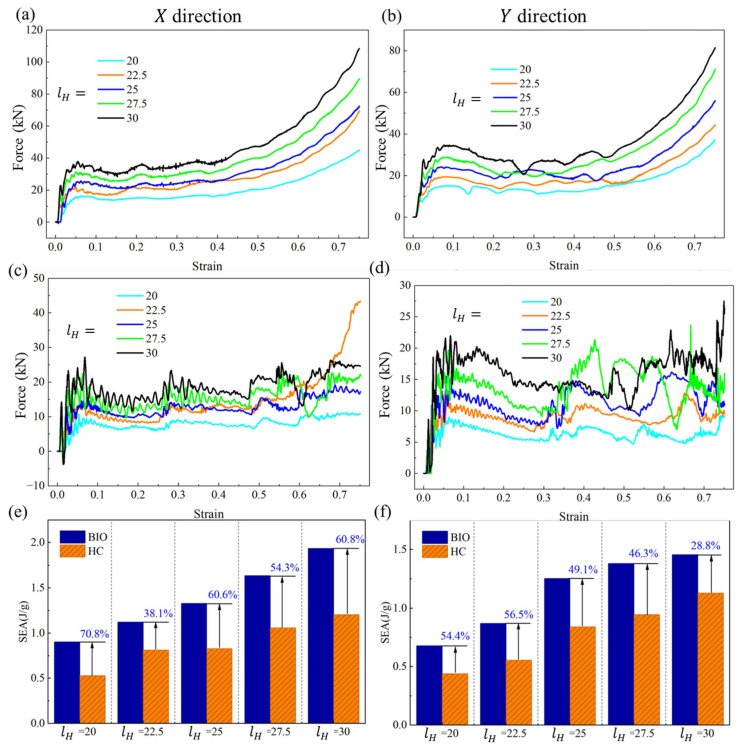
Significant in-plane performance improvement: (**a**) the biomimetic honeycombs under X-direction loading; (**b**) the biomimetic honeycombs under Y-direction loading; (**c**) the conventional honeycombs under X-direction loading; (**d**) the conventional honeycombs under Y-direction loading; (**e**) comparison of in-plane SEA in the X-direction; and (**f**) comparison of in-plane SEA in the Y-direction.

To further investigate whether the proposed biomimetic spiral-reinforced design can improve the weak direction of conventional honeycombs, the in-plane compressive performance was obtained using the simulation method, as shown in Figure 8. The biomimetic structures considered here are the same as those in the previous section, namely specimens with a wall thickness of 2 mm and different wall lengths. For each biomimetic configuration, a conventional hexagonal honeycomb with the same mass, volume, and wall length was correspondingly established, so that the reinforcing effect introduced by the spiral substructures could be evaluated on a comparable basis.

Figure 8 summarizes the in-plane compressive responses of the two types of honeycombs. Figure 8a,c,e correspond to the X-direction results, while Figure 8b,d,f show the Y-direction results. In the X-direction, the SEA of the biomimetic honeycomb reaches 0.90 J/g, which is 70.8% higher than that of the conventional honeycomb (0.53 J/g) when $l_{H}=20\;mm$, as shown in Figure 8e. In the Y-direction, the corresponding improvement is 54.4%, as shown in Figure 8f. Such a pronounced in-plane improvement indicates promising potential for more design, e.g., sandwich structures, because this is consistent with related studies like foam-filled sandwich reported by Eyvazian et al. [33,34,35], where energy absorption improvements benefited from core enhancement of up to 80%. In summary, under equal-mass and equal-volume conditions, the proposed biomimetic design can significantly enhance the in-plane energy absorption of the honeycomb in both directions. It demonstrates that the spiral reinforcement is not effective only for one specific in-plane direction, but can provide a broader multidirectional strengthening effect. A further comparison between the two in-plane directions shows that the enhancement in the X-direction is generally more pronounced than that in the Y-direction. This suggests that the spiral-reinforced configuration interacts differently with the original cell topology under different in-plane loading paths.

Overall, the results clearly show that the proposed biomimetic spiral-reinforced honeycomb can markedly improve the weak in-plane performance of the conventional honeycomb. It should also be noted that, although the in-plane performance is significantly enhanced, it remains lower than the out-of-plane performance. Therefore, the present study only proposes a feasible biomimetic strategy to alleviate the discrepancy between their in-plane and out-of-plane responses and thereby improve their integrated mechanical performance under complex loading conditions.

### 5.3. Underlying Mechanism for the Integrated Enhancement

**Figure 9:**
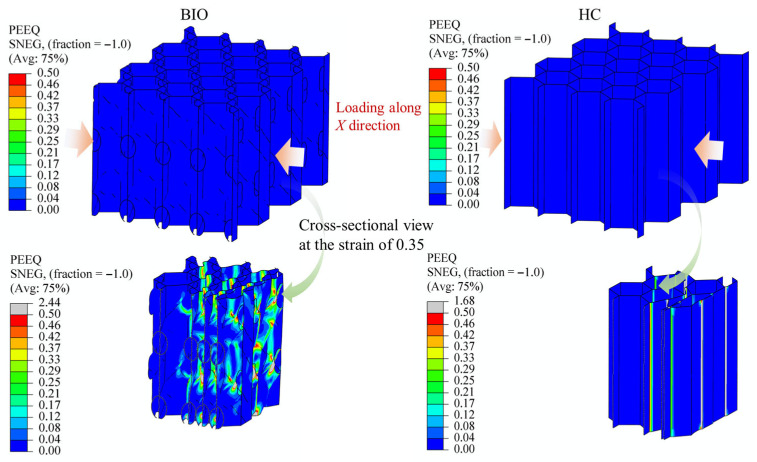
Comparison of the plastic strain regions between the biomimetic honeycomb and the conventional honeycomb under X-direction loading, shown at a global compressive strain of 0.35. Straight arrows indicate the loading direction, while curved arrows represent the source of the section.

**Figure 10:**
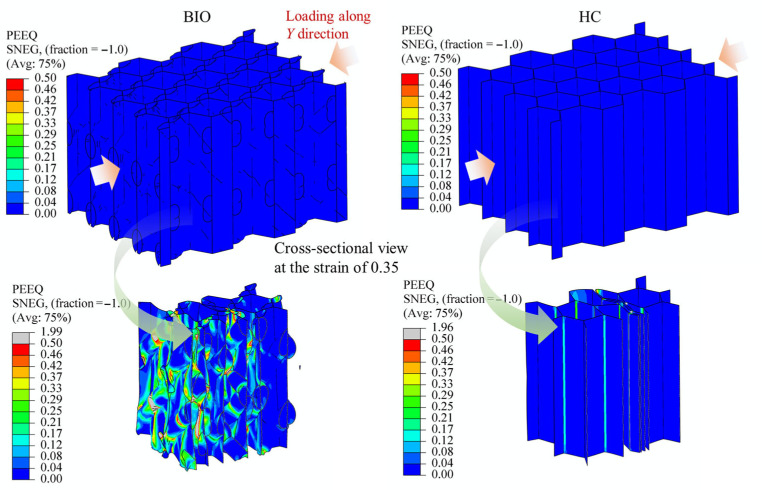
Comparison of the plastic strain regions between the biomimetic honeycomb and the conventional honeycomb under Y-direction loading, shown at a global compressive strain of 0.35. Straight arrows indicate the loading direction, while curved arrows represent the source of the section.

**Figure 11:**
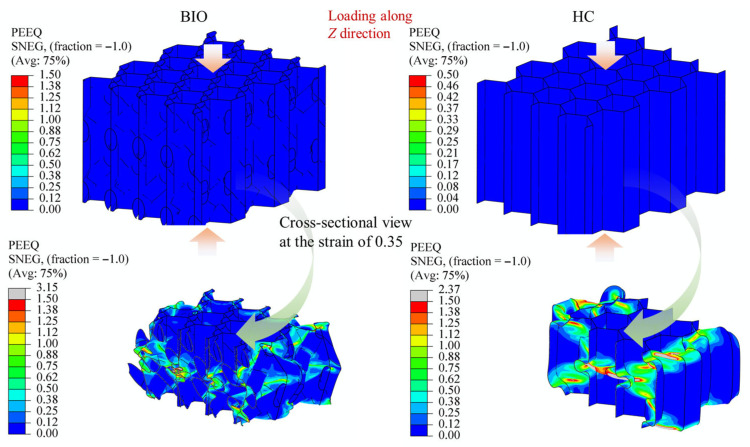
Comparison of the plastic strain regions between the biomimetic honeycomb and the conventional honeycomb under Z-direction loading, shown at a global compressive strain of 0.35. Straight arrows indicate the loading direction, while curved arrows represent the source of the section.

For honeycomb-type energy-absorbing structures, a high energy-absorption capacity is generally associated with three characteristics, namely a wide distribution of the plastic deformation region, weak strain localization, and relatively uniform stress within the whole structure. Therefore, the essence of improving the integrated energy-absorption performance lies in promoting a broader and more plastic deformation process throughout the structure. In this work, the plastic strain contours (PEEQ) under the X-, Y-, and Z-direction loadings clearly support this mechanism, as shown in Figure 9, Figure 10 and Figure 11. Since the proposed structure is periodic, the deformation behavior of a single cell is representative of the overall structural response. The plastic strain contours (PEEQ) of locale cells presented below thus reflect the cell-level deformation characteristics under each loading direction.

Under X-direction loading, the conventional honeycomb exhibits a strongly localized deformation pattern, as shown in Figure 9. The plastic strain is mainly concentrated on the connecting hinges between the thin-walled sections, while most regions remain at a relatively low plastic level. This indicates that the load is preferentially transmitted through weak paths, i.e., the hinges, resulting in early local instability. In contrast, the biomimetic spiral-reinforced honeycomb shows a much broader plastic strain distribution. The high-strain regions are not confined to hinges, but are distributed over cell walls and spiral parts. The applied load was redistributed to broader regions, thereby weakening strain localization and promoting a more sufficient energy absorption. This is consistent with the much higher in-plane force level and SEA obtained in the X-direction. In addition, a similar phenomenon can also be observed under Y-direction loading, as shown in Figure 10.

In both cases of X- or Y-direction loading, the plastic regions are broadly distributed, the localization degree is reduced, and the spiral parts participate significantly in the deformation process. The reason is that the point-symmetric double-spiral arrangement inside each cell provides oblique load-transfer pathways in both in-plane directions, so that the applied load can be redistributed from the initially compressed walls to neighboring cell walls and internal reinforcements under either X- or Y-direction loading. As a result, the in-plane energy-absorption capacities in the two directions become much higher than those of the conventional honeycomb.

As shown in Figure 11, the Z-direction results further explain why the proposed design can improve the integrated performance without significantly sacrificing the out-of-plane performance. Under out-of-plane compression, both structures show a typical global crushing mode, and the plastic deformation extends over a relatively large region. This is the intrinsic advantage of the conventional honeycomb in the out-of-plane direction. However, compared with the conventional honeycomb, the biomimetic structure still exhibits a more dispersed plastic strain distribution in the cross-sectional view. In the conventional honeycomb, the high plastic strain is mainly concentrated around the folding hinges of the cell walls. In the biomimetic structure, besides the folding hinges, the internal spiral parts also display evident plastic deformation and participate in the energy absorption. Therefore, the introduced spiral reinforcements do not destroy the original out-of-plane energy absorption mechanism of the honeycomb, but instead provide additional energy-dissipation regions. This is why the proposed structure can preserve an out-of-plane performance comparable to that of the conventional honeycomb.

The above results indicate that the key role of the spiral-reinforced design lies in its ability to reconstruct the internal load-transfer mode of the honeycomb. In the proposed biomimetic structure, the embedded spiral members act as internal bridging and transfer paths. Once the external load is applied, part of the load is transmitted along the spiral trajectories to adjacent walls and neighboring cells. Meanwhile, because the spiral parts are inclined with respect to the principal loading directions, they can deform through a combination of bending, stretching, and torsion, instead of simple local bending alone. This makes it possible to transform a highly localized collapse mode into a more distributed mode. The plastic strain contours provide direct evidence for this process, since pronounced plasticity develops continuously along the spiral parts and the connected cell walls under all three loading directions.

## 6. Conclusions

A biomimetic spiral-reinforced honeycomb was proposed by embedding double-spiral substructures into a conventional hexagonal honeycomb. Through quasi-static compression tests and validated finite element simulations, the multidirectional energy absorption behavior and the associated enhancement mechanism were investigated. The main conclusions are as follows:

(1) The proposed structure retains the favorable out-of-plane performance of the conventional honeycomb, but the reduction in out-of-plane SEA remains limited.
(2) The weak in-plane performance is significantly improved by the spiral-reinforced design. The maximum SEA enhancement reaches 70.8% in the X-direction and 56.5% in the Y-direction.
(3) The performance improvement is mainly attributed to the additional oblique load-transfer paths introduced by the spiral parts, which promote broader plastic-zone development and reduce strain localization.
(4) The proposed bio-design provides an effective route to narrow the gap between in-plane and out-of-plane performance, thereby improving the integrated mechanical performance under complex loading conditions.

It should be noted that the current study relies on FDM-based 3D printing for specimen fabrication, which is well-suited for geometric flexibility and rapid prototyping but may limit large-scale manufacturing feasibility due to relatively high production costs. Future work could explore metal-based manufacturing routes to extend the applicability of the proposed design. For instance, aluminum beetle elytron plates with similar bioinspired configurations have been fabricated via aluminum strip pressing and bonding methods [36], demonstrating the feasibility of low-cost metallic fabrication for biomimetic thin-walled structures. In addition, the limited number of specimens (*n* = 2) represents a statistical limitation of the present study, and future work with larger sample sizes is recommended for more design and development.

## Figures and Tables

**Table 1:** PLA material parameters (averages derived from anisotropic test results).

Young’s Modulus	Poisson’s Ratio ^1^	Yield Strength
910 MPa	0.35	25 MPa

^1^ It is referenced from [32].

## Data Availability

The original contributions presented in this study are included in the article. Further inquiries can be directed to the corresponding author.

## Abbreviations

The following abbreviations are used in this manuscript:

BIO	It refers to the biomimetic structure proposed in this study
HC	It refers to the traditional hexagonal honeycomb structure
SEA	It refers to the energy absorbed per unit mass of the material
